# Dynamic analysis of the subsea production system with lazy-wave risers attached to FPSO

**DOI:** 10.1371/journal.pone.0291603

**Published:** 2023-09-15

**Authors:** Dapeng Zhang, Bowen Zhao, Keqiang Zhu, Haoyu Jiang

**Affiliations:** 1 Ship and Maritime College, Guangdong Ocean University, Zhanjiang, Guangdong, China; 2 Ocean College, Zhejiang University, Zhoushan, Zhejiang, China; 3 Faculty of Maritime and Transportation, Ningbo University, Ningbo, Zhejiang, China; 4 School of Electronics and Information Engineering, Guangdong Ocean University, Zhanjiang, Guangdong, China; University of Zanjan, ISLAMIC REPUBLIC OF IRAN

## Abstract

The lazy-wave riser is an input and output riser for a flexible development system, which is widely used in all the riser and pipeline systems. Because of the influence of various factors, its configuration description, control and motion which have a strong nonlinear character are complex during the running process of the lazy-wave riser. Reference to the specific structure and environmental parameters of a certain lazy-wave risers system with a 300 thousand tons FPSO, with the basis of the specific process of the flexible riser system at work, the lazy-wave risers were discretized into lumped mass models, combined with AQWA, the simplified dynamic model of the whole system at the depth of 2100m has been established by the large hydrodynamic analysis software OrcaFlex. The dynamic response characteristics of the lazy-wave risers have been given by using time domain coupling method. With and without the consideration of the 2nd wave drift load in the simulation process, the effects of the 2nd wave drift load on the results are obtained. The simulation results reveal the difficulty of simulation convergence caused by a large number of risers and flexible components. The 2nd order wave drift loads have a significant effect on the riser system, resulting in the increasement of the effective tension at each end of each riser. To counteract the magnitude of the FPSO response caused by such loads, the number of mooring lines needed to be increased or combined with dynamic positioning techniques to optimize the design.

## Introduction

Exploration and drilling of oil and gas have extended and moved from offshore to deep (>300 m) and ultra-deep (>1500 m) water areas, due to increasing demand of energy. Floating production storage and offloading (FPSO), spar, tension leg platform (TLP) and semi-submersible (SEMI) are popular production systems. These systems usually consist of a hull, a mooring system and risers.

Under the external load, the dynamic responses of underwater production systems at mooring state have been a hot issue among scholars, for example, Wichers and Dev [1] derived the motion equations of FPSO and its mooring lines in time domain based on the conventional uncoupled theory. The results showed that the only the complete coupling of FPSO and risers system can the realistic system design values be obtained. Tahar and Kim [2] developed a computer program for coupled dynamic analysis of a tanker-based turret-moored FPSO in waves, non-parallel winds and currents. In the computer program, the floating body was modeled as a rigid body with six degrees of freedom. The conclusion that the use of full second-order force quadratic transfer function (QTFs) increased sway and yaw rms (slowly varying) responses provided a reference for wave forces study in this paper. Baar et al. [3] also observed that the extreme response of a turret-moored FPSO was sensitive to the non-collinear environmental conditions of waves, current and wind and the location of the internal turret had influence on the motion response and tension of the mooring lines. Tahar and Kim [4] developed numerical solutions for the coupled-dynamic analysis of a deep-water platform with polyester mooring lines, and then these numerical results were compared with those with equivalent elastic lines. The simulation results proved the reliability of numerical solutions. Ji et al. [5] investigated the dynamic response characteristics of a single point tur-ret-moored FPSO along with middle water arch system under the combined parallel wave, wind and current conditions by using coupled time domain analysis code AQWA. The top tensions of mooring cables at the different sides of FPSO were obviously smaller than that of single body model, however the tensions of mooring cables at the same side of the FPSO were magnified due to the tension reduction of risers. This conclusion benefits the design of mooring cables at different sides of FPSO. Gam et al. [6] proposes a dynamic analysis procedure in the time domain and the frequency domain to solve the nonlinearity issue. Introducing a linearization coefficient for the drag term of the Morison equation benefited the calculation of wave forces. Yang et al. [7] studied the dynamic responses of a truss spar and its mooring line system in the time domain. From the coupled analysis results, it could be found that the dynamic coupling effects played an important role in deep water especially when the response amplitude was very large. The effects of mooring inertia and damping were important for the dynamic analysis of a deep-water compliant platform.

According to the literature, in the time domain coupling calculation of subsea production systems, many scholars usually ignored the influence of riser system. In fact, the riser system also affects the stiffness of the whole mooring system because the marine riser is a key component of deep-sea floating structures. It is not only a connecting channel between sea surface and seabed but also a link between underwater wellhead and floating body [8]. In recent years, some scholars considered the coupling between ships and mooring risers and regarded the action of risers as the tension of the ship hull. Arcandra [9] investigated the hydrodynamic properties of platforms, mooring cables and risers. The floating platform was modeled as a rigid body with six degrees of freedom and the mooring cable dynamics were modeled using a rod theory and finite element method, which was still popular at present. Kim et al. [10] established a coupling dynamic model of the hull, mooring cables and riser system of a turret FPSO and made an analysis in the time domain. Notably, the dynamic mooring tension could be underestimated with mooring system when mooring dynamic effects were significant. Sun et al. [11] investigated the effects of the riser parameters and the floating buoy to the flexible risers. The results showed that the increase in riser wall thickness would lead to a non-linear reduction in riser stress. Lopez et al. [12] studied the motion response of the FPSO with mooring and riser system for both the Full load and the Ballast load conditions. The motion response spectra analysis revealed that risers had a great influence on low-frequency damping, particularly in the surge direction, whereas the damping mainly contributes to roll of the wave frequency motion response. These studies all proved that coupled with the mooring system and riser system, the FPSO exhibits strong nonlinear characteristics, so the time-domain coupling method is usually needed for accurate simulation.

At present, the commonly layout forms used for deep-water flexible risers are Lazy-S configuration, steep-S configuration, Lazy-wave configuration and steep-wave configuration [13]. In all the layout forms, the Lazy-wave configuration improves anti-fatigue performance and decrease the effect of the riser, which can absorb the upper platform motion very well and have a good protection for the touchdown zone, and make the system a better compliance and minimize the transmission of hull motions. At the same time, it can better conform to the drift and heave motion of the floating body and then improve the dynamic response characteristics of the riser. Moreover, this characteristic is more obvious with the increase of water depth [14].

Up to now, many scholars have done a lot of meaningful research on the Lazy-wave configuration of deep-water flexible risers, as well as other layout forms. Yang et al. [15] presented some results from a comprehensive study on the fatigue life prediction of Lazy-wave riser and illustrated the sensitivity of the risers’ fatigue to the vessel motions, the drag coefficient along the risers, the structural damping and the seabed stiffness. The analyses indicated the strong dependence of the riser fatigue life on these parameters. Based on the conventional small deformation beam theory for the flowline lying on the seabed and coupled with a large deformation beam theory for the suspended section, Wang et al. [16–18] proposed a nonlinear model for deep-water steel Lazy-wave riser configuration to simulate the suspended part of the riser. They also investigated the influences of ocean current, internal flow and deep-water on the static performance of the riser [19–21]. The proposed nonlinear model and numerical results were of basic and important reference value for the design and dynamic analysis of deepwater steel lazy-wave riser installation. Santillan et al. [22] presented a systematic study on the Lazy-S and steep-S shaped risers by simulating the buoyancy segments as a concentrated lift force applied at the arch bend peak point. The effects of the length of the buoyed section, length of the upper unbuoyed section, magnitude of the buoyancy force, and velocity of the steady horizontal current on the equilibrium shape were investigated. The results showed that as the current velocity was increased from zero, the frequency of the riser initially decreases and then increases. Kim et al. [23] used a hull-mooring-riser fully-coupled dynamic program to analysis the dynamic and structural performances of a deep-water turret-moored FPSO in 100-yr hurricane condition. The lazy-wave steel catenary riser (LWSCR) could be considered as a good solution for deep-water FPSO in extreme environments. Yue et al. [24] performed analytic study to compare structural performance of conventional steel catenary riser (SCR), shaped steel catenary riser, and lazy-wave steel catenary riser, which were attached to a turret moored FPSO in 800 m water depth. In the study, the Von-Mises stress and fatigue life of the three types were directly compared. The results indicated that traditional LWSCR showed sufficient fatigue life, provided that the vessel headings were controlled within a range of angles, while more measures were needed for the Shaped SCRs to have enough fatigue life. Cheng et al. [25] carried out a Lazy-wave model scale test to explore the hydrodynamic response of a steel Lazy-wave riser under forced oscillatory and random motions in a wave basin. The experimental results also provided the validation model for this paper. Ruan et al. [26–28] analyzed the static behavior of deep-water Lazy-wave risers on the elastic seabed and estimated the effective static stress range with vessel slow drift motion. They drew the conclusions that hang-off inclination angle had a significant influence on the mechanical behavior of lazy-wave configuration, which provided reasonable guidelines for engineering application of installation. However, they also pointed out that further research needed to be carried out on dynamic response of deep water lazy-wave riser. Ai et al. [29], Hu et al. [30], Oh et al. [31] and Trapper [32] used the numerical methods to study the response characteristics of the Lazy-wave risers in offshore, deep and ultra-deep water areas, respectively. Cheng et al. [33] proposed a numerical model based on three-dimensional large deformation rod theory. The whole space model was divided into four parts: touchdown segment, decline segment, buoyancy segment and hang-off segment to accurately simulate the favorable motion performance of the Lazy-wave riser, involving the effects of vessel motion, wave-current loads, riser-seabed interaction and internal flow. Amaechi et al. [34] carried out a series of research work on hydrodynamic characteristics, mechanical behavior and sensitivity analysis of risers attached to the catenary anchor leg moorings (CALM) buoys. Parametric studies on Lazy-S configuration, which can be useful in offshore industry with the effect of current on the offloading system.

In the study of the hydrodynamic characteristics of the risers, the large displacement tonnage and the extremely complex system make the experiment very difficult. The flow characteristics and the vibration of the riser can also bring uncertainty to experimental research [35]. Therefore, numerical simulations are usually used for such super large systems [36, 37]. Amaechi et al. [38–45] made tremendous numerical efforts as well as experimental studies, and achieved plentiful and substantial results, coving composite marine risers for floating and fixed platforms, dynamic responses of CALM moored tanker system, optimization of mooring line design parameters and the fluid-structure interaction. They contributed to the research of dynamic analysis of marine risers attached to offshore floating and fixed platforms.

According to the literature, there are few reports on the dynamics analysis between a large number of risers and floating structures. With the increase of the depth of the water and the increase of the number of risers, the nonlinearity of the system is also increased, and the static equilibrium of the risers is difficult to capture and the coupling between the upper floating structure and the risers, especially in the ultra-deep water conditions. For the underwater production system with large displacement and large number of system flexible components which is running in ultra-deep water areas, it is a great challenge to make simulation convergence, to some extent, it is also a great innovation in engineering application. The novelty of this paper lies in the numerical simulations, phenomenon observation and dynamic analysis of a large number of risers attached to FPSO. Reference to the specific structure and environmental parameters of a certain 55 Lazy-wave risers system with a 300 thousand tons FPSO, the 55 Lazy-wave risers were discretized into lumped mass models. A simplified numerical model of the whole system at the depth of 2100m has been established by a software OrcaFlex. The dynamic response characteristics of the Lazy-wave risers have been given by using time domain coupling method. With and without the consideration of the 2nd wave drift load in the simulation process, the effects of the 2nd wave drift load on the results have been obtained. Combined with the calculation results, the optimal design scheme of the riser system is given, which has certain guiding significance for the specific engineering practice.

## Computational theory

### Catenary method for static equilibrium of marine pipeline

No matter what kind of layout of the marine pipeline, the static analysis should be carried out in numerical simulation, which is used for achieving two purposes: firstly, it is to determine the equilibrium shape of the marine flexible riser system under the combined action of gravity, buoyancy and hydrodynamic drag force; secondly, it can provide the initial pipeline configuration form for the dynamic analysis of the system.

The full static analysis is a linear static calculation that includes all forces. In particular, it also includes the action of the bending stiffness and the interaction between the two-dimensional components. Those are the forces that cause large impact loads at the initial stage of simulation, which are omitted in the catenary method. As the full static calculation method includes these effects, therefore, OrcaFlex recommends the use of full static analysis in most cases.

Static analysis is required when using full static analysis. The second step is to fill the linear data table completely. It uses the specified static analysis method to obtain the initial configuration of the required pipeline, and then it can seek a balance posture. So, in order to set up the data in static analysis step 1, we need to give a reasonable initial configuration, and select catenary method, specifying method, fast method or spline method as one choice.

The default setting option is the catenary method under full static analysis. Usually, this is a better choice, because the catenary method is faster and can provide more accurate estimation of the equilibrium configuration in many cases. It also provides a better starting point for full static analysis and computation.

According to whether the bending stiffness of the pipeline is considered in the calculation process, the catenary method can be divided into the free hanging catenary method and the rigid catenary method. In the free hanging catenary method, the bending stiffness of the pipeline is neglected, the analytical solution is obtained by the equilibrium differential equation of the pipeline element, then the configuration of the pipeline can be obtained by several iterative iterations. The free hanging catenary method is generally considered to be applied to the calculation of pipelines in the deep sea; the rigid catenary method has high calculation accuracy, but its calculation process is complex (considering the bending stiffness of the pipeline), which cannot solve the equilibrium differential equation directly and it is necessary to use iterative method to guarantee the convergence. But a lot of researches have proved that with the depth increasing, the natural catenary method could better meet the computing requirements and also can save a lot of time. As the water depth has reached 2100m, so this paper uses the natural catenary method to calculate the static equilibrium of the flexible riser.

**Fig 1:**
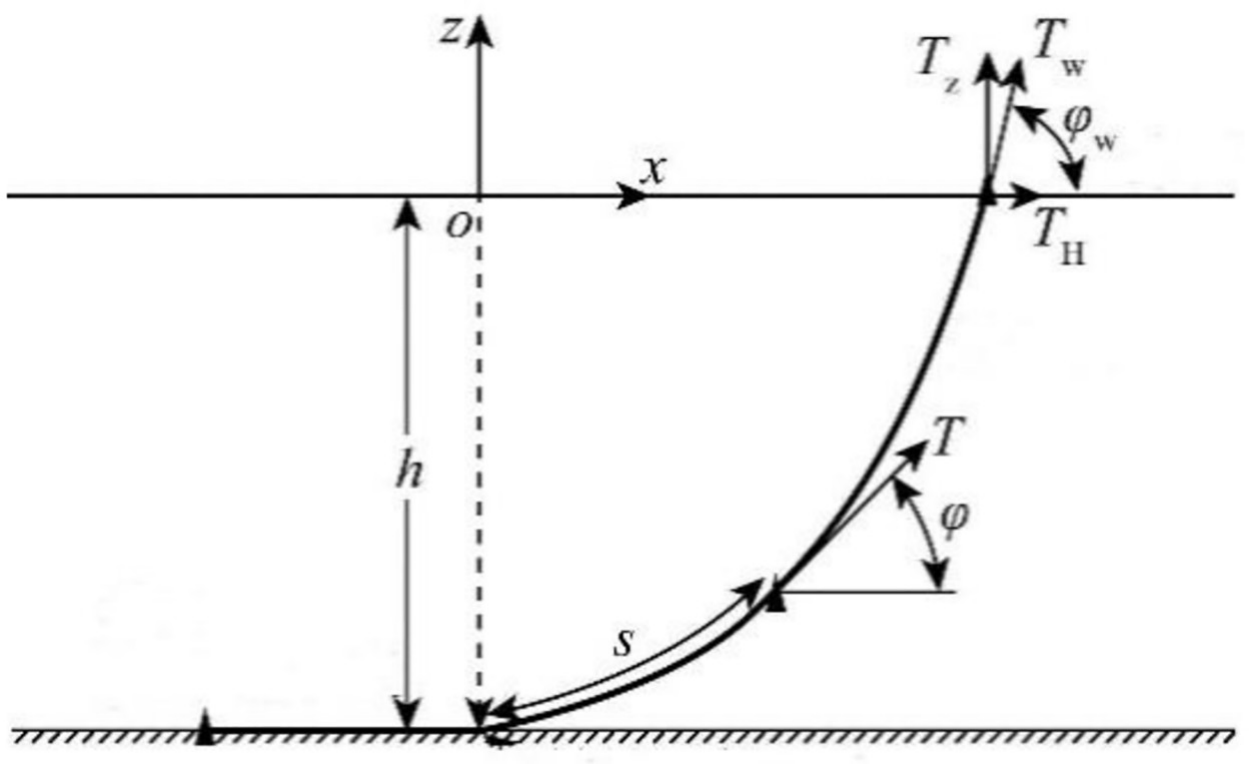
The configuration of the free hanging catenary layout form.

**Fig 2:**
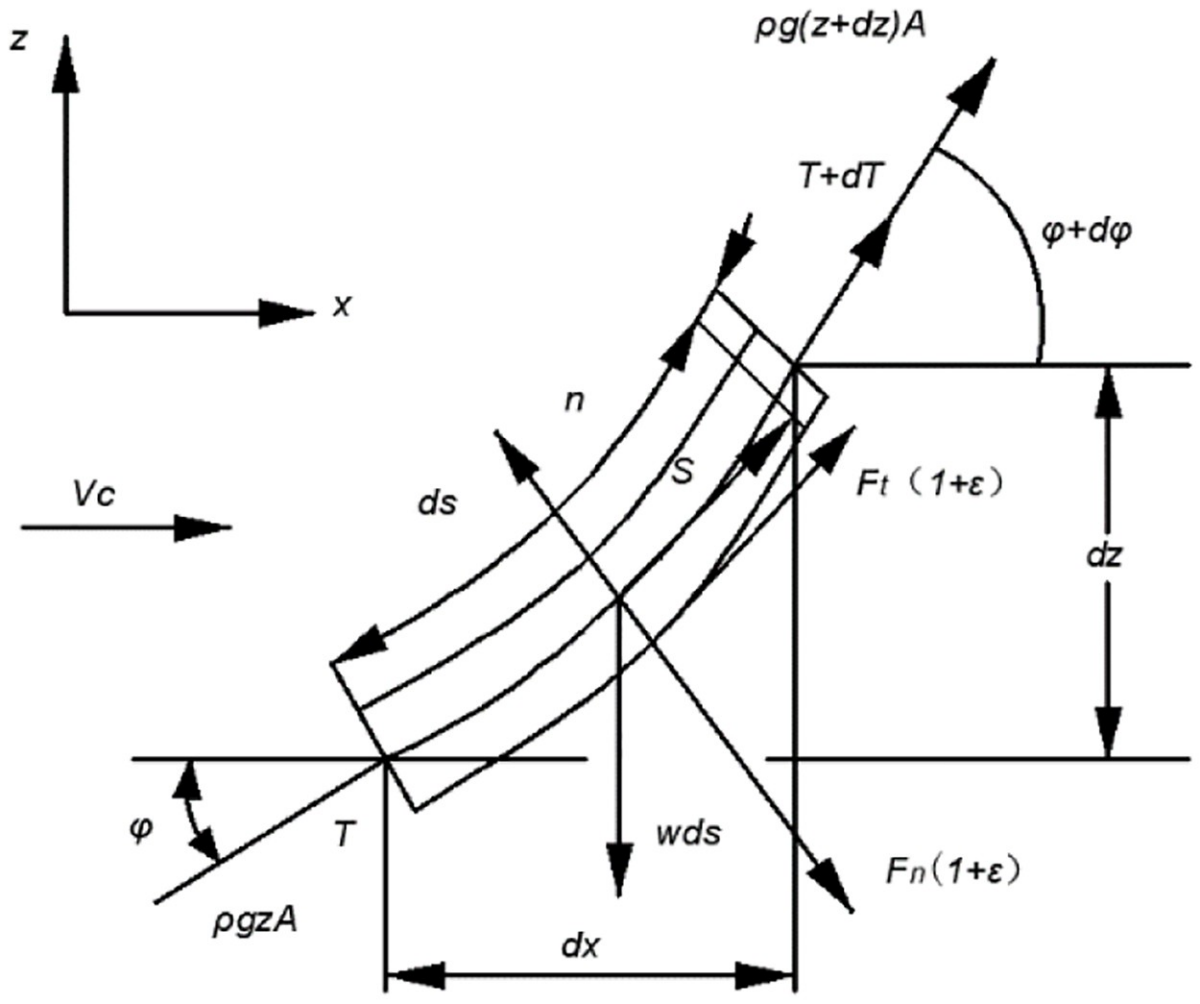
Force analysis of a catenary micro section.

Compared with other layout forms, the free hanging catenary configuration is the simplest and the most economical layout form. It is generally believed that the spatial configuration of free suspension flexible risers can be approximately represented by the catenary equation without considering the bending stiffness. Here, the catenary equation will be deduced preliminarily. Fig 1 is the schematic diagram of the free hanging catenary layout. Fig 2 depicts the force analysis a catenary micro section.

In order to simplify the derivation process, the influence of inertia force and fluid force is not taken into consideration. Under this assumption, the static equilibrium equation of a single catenary can be deduced as follows:

With the basis of the force analysis of the catenary micro section and the normal and axial stress equilibrium relationship of the pipeline, the following expression can be obtained:

dT−ρgAdz−Wsinφds=0 (1)

Tdφ−ρgzAdφ−Wcosφds=0 (2)

As *T*′ = *T* − *ρgzA*, so

$$dT^{\prime }=W\;\text{sin}\;\varphi ds$$ (3)

$$T^{\prime }d\varphi =W\;\text{cos}\;\varphi ds$$ (4)

With further transformation of the above two formulas, the following equation can be obtained:

$$T_{H}=T^{\prime }\;\text{cos}\;\varphi ,\frac{T_{H}}{\text{cos}\;\varphi }d\varphi =W\;\text{cos}\;\varphi ds$$ (5)

Then we can get the following equation by integration of Eq (5):

$$s-s_{0}=\frac{T_{H}}{W}\int_{\varphi_{0}}^{\varphi }\frac{1}{{\text{cos}}^{2}\;\theta }d\theta =\frac{T_{H}}{W}\;\text{tan}\;\varphi$$ (6)

As *dx* = *ds* cos *φ*, so

$$x\text{-}x_{0}=\frac{T_{H}}{W}\int_{\varphi_{0}}^{\varphi }\frac{1}{\text{cos}\;\theta }d\theta =\frac{T_{H}}{W}\;\text{ln}\;\frac{1+\text{sin}\;\varphi }{\text{cos}\;\varphi }$$ (7)

As *dz* = *ds* sin *φ*, the difference of *z*-*z*_0_ can be expressed as:

$$z\text{-}z_{0}=\frac{T_{H}}{W}\int_{\varphi_{0}}^{\varphi }\frac{\text{sin}\;\theta }{{\text{cos}}^{2}\;\theta }d\theta =\frac{T_{H}}{W}(\frac{1-\text{cos}\;\varphi }{\text{cos}\;\varphi })=z+h$$ (8)

$$\text{sinh}\frac{xW}{T_{H}}=\text{tan}\;\varphi ,\text{cos}\;\text{h}\frac{xW}{T_{H}}=\frac{1}{\text{cos}\;\varphi }$$ (9)

Combined with Eqs (8) and (9), we can get the expression of *z*+*h*:

$$z+h=\frac{T_{H}}{W}\left[\text{cosh}(\frac{W}{T_{H}}x)-1\right]$$ (10)

Where, $\frac{T_{H}}{W}=s\;\text{cot}\;\varphi_{w}$.

As a result, z can be written as the following:

$$z=s\;\text{cot}\;\varphi_{w}\left[\text{cosh}(\frac{x}{s\;\text{cot}\;\varphi_{w}})-1\right]-h$$ (11)

When we know the depth *h*, the length of the flexible riser *s* and the hanging off angle *φ*_*w*_, we can get the spatial coordinate position and the configuration of the riser by the equations, and based on the above calculation results, the static equilibrium of the flexible riser can be obtained, then the static configuration can be given. As the Lazy-wave riser can be divided into several catenary segmentations, then the whole static configuration of the Lazy-wave riser can be obtained with the method of the catenary segmentations, which will be introduced in the following in detail.

### Solution for the static equilibrium of the Lazy-wave riser

**Fig 3:**
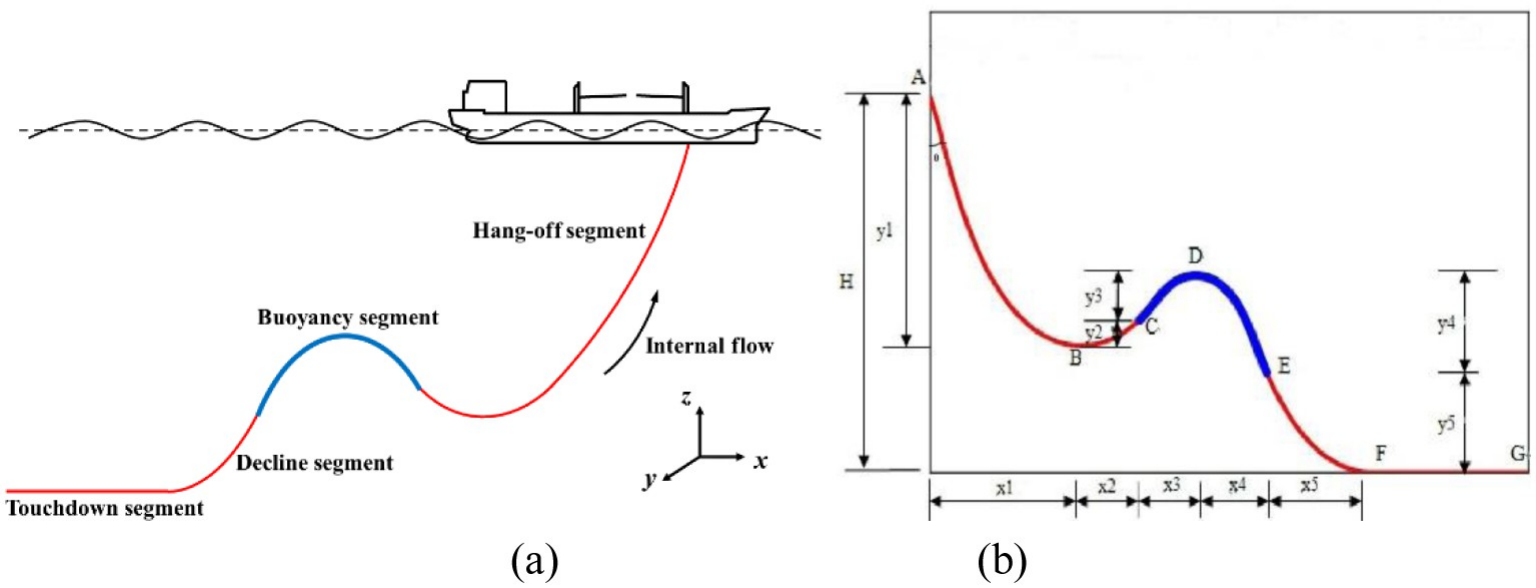
(a) The global configuration of the Lazy-wave riser; (b) The local configuration of the Lazy-wave riser.

**Fig 4:**
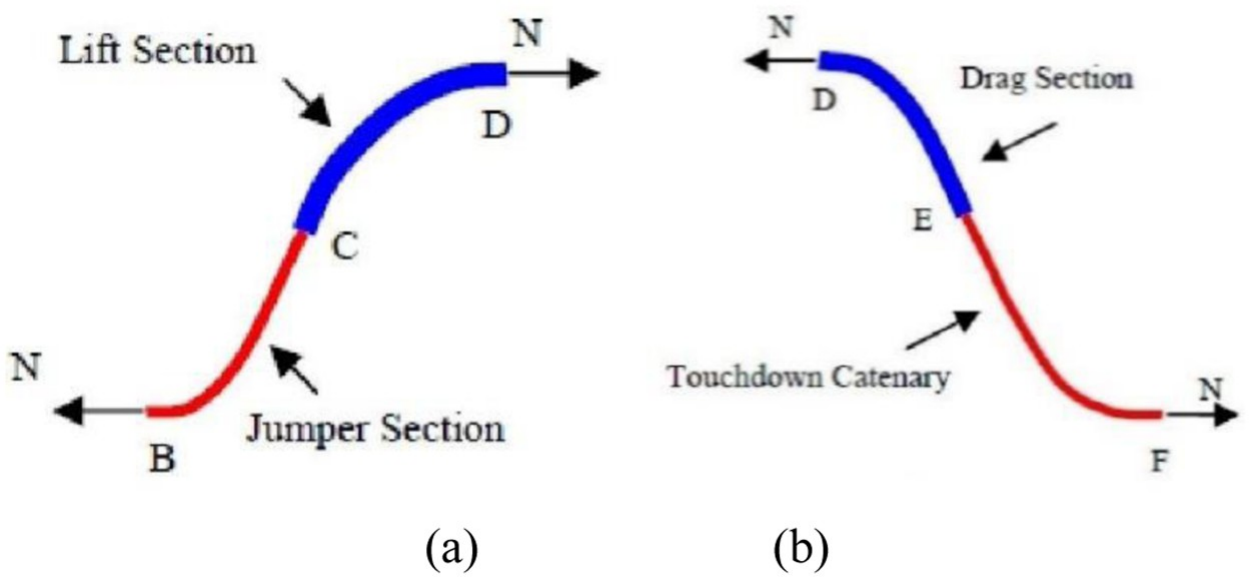
(a) The lift section and jumper section; (b) The drag section and touchdown catenary.

The spatial configuration of the Lazy-wave riser is shown in Fig 3, where the BF segment can be further divided into the two segments, just as shown in Fig 4.

As shown in Fig 4, the Lazy-wave riser is a common line for risers and this configuration has more floating buoy or some other buoyancy devices than catenary. Through the static analysis of the Lazy-wave riser, it can be seen that the direction of the joint force acting on B point and D point in is horizontal and the numerical value is equal to the horizontal force F at touchdown point N. There is no vertical shear force at B point and D point, so the gravity of BC segment is equal to the buoyancy of CD segment, the buoyancy of DE segment equals the gravity of EF segment. Through the above analysis, AB segment, BC segment, CD segment, DE segment and EF segment can be considered as catenary. Therefore, in the local coordinate system (see Fig 4), the hanging off section AC, the floating section CE and the descent segment EF can be expressed by the theoretical formula [26, 46], which is used for the Lazy-wave riser:

$$y=a_{i}\left(\text{cosh}\frac{x}{a_{i}}-1\right)=\frac{a_{i}}{2}\left({\text{e}}^{\frac{x}{a_{i}}}+{\text{e}}^{-\frac{x}{a_{i}}}-2\right)$$ (12)

$$v=a_{j}\left(\text{cosh}\frac{p}{a_{j}}-1\right)=\frac{a_{j}}{2}\left({\text{e}}^{\frac{x}{a_{j}}}+{\text{e}}^{-\frac{x}{a_{j}}}-2\right)$$ (13)

$$q=a_{k}\left(\text{cosh}\frac{p}{a_{k}}-1\right)=\frac{a_{k}}{2}\left({\text{e}}^{\frac{x}{a_{k}}}+{\text{e}}^{-\frac{x}{a_{k}}}-2\right)$$ (14)

Where, *a*_*i*_, *a*_*j*_, *a*_*k*_ are the curvature of *B*, *D*, *E* in local coordinate system, respectively.

It is assumed that the mass per unit length of the hanging off segment, the floating section and the descent section are *m*_*i*_, *m*_*j*_ and *m*_*k*_, respectively; therefore, the wet weight of each section flexible riser is

$$Q_{i}=m_{i}g,Q_{j}=F-m_{i}g,Q_{k}=m_{k}g$$ (15)

Where, *F* is the upward buoyancy provided by the floating section of the unit length. When *m*_*i*_ = *m*_*k*_, which means *Q*_*i*_ = *Q*_*k*_ = *Q*. Here, the buoyancy factor is defined as the following:

$$n=\frac{F-Q}{Q}$$ (16)

Where, *n* is the buoyancy factor. From the calculation formula of buoyancy factor, we know that it is a dimensionless parameter.

According to the catenary equation, the jumper section and the lift section can be expressed as:

$$S_{2}=a_{i}\;\text{sinh}\;\frac{x_{2}}{a_{i}},S_{3}=a_{j}\;\text{sinh}\;\frac{x_{3}}{a_{j}}$$ (17)

From the above analysis, we can obtain the following equation:

$$S_{2}Q_{i}=S_{3}Q_{j},\frac{S_{2}}{S_{3}}=\frac{Q_{j}}{Q_{i}}$$ (18)

Then the following expression can be deduced:

$$\frac{S_{2}}{S_{3}}=\frac{x_{2}}{x_{3}}=\frac{Q_{j}}{Q_{i}}=\frac{a_{i}}{a_{j}}$$ (19)

Similarly, for the drag section *DE* and the descent section *EF*, there are also similar equations as follows:

$$\frac{S_{4}}{S_{5}}=\frac{x_{4}}{x_{5}}=\frac{y_{4}}{y_{5}}=\frac{Q_{k}}{Q_{j}}=\frac{a_{j}}{a_{k}}$$ (20)

For a Lazy-wave riser, the structural distribution of the entire flexible riser can be calculated when the length *S*_*i*_ of the hanging off section, the length *S*_*j*_ of the floating section and the length *S*_*k*_ of the descent section are given. Just as can be seen from Fig 3:

$$S_{i}=S_{1}+S_{2},S_{j}=S_{3}+S_{4},S_{k}=S_{5}$$ (21)

The length of the whole Lazy-wave riser (without the touchdown section) is: *S* = *S*_*i*_ + *S*_*j*_ + *S*_*k*_, combined with the above equation and the Eq (19), the length of the hanging off section can be obtained:

$$S_{1}=S-(1+\frac{Q_{j}}{Q_{i}})S_{j}$$ (22)

As the hanging off section is the catenary structure, so the length of the hanging off section can be expressed as:

$$S_{1}=a_{i}\;\text{cot}\;\theta$$ (23)

Where, *θ* is the top suspension angle, as shown in Fig 3. By substituting it into (22), the curvature of the touchdown point can be obtained:

$$a_{i}=[S-(1+\frac{Q_{j}}{Q_{i}})S_{j}]\;\text{tan}\;\theta$$ (24)

And the horizontal span and height of the hanging off section can be expressed as:

$$x_{i}=a_{i}\;\text{arcsin}\;h(\text{cot}\;\theta ),y=a_{i}(\text{cosh}\frac{x_{1}}{a_{i}}-1)$$ (25)

Similarly, the horizontal span and height of the jumper section can be obtained:

$$x_{2}=a_{i}\;\text{arcsin}\;h(\frac{S_{i}-S_{1}}{a_{i}}),y_{2}=a_{i}(\text{cosh}\frac{x_{2}}{a_{i}}-1)$$ (26)

The horizontal span and height of the lift section:

x2=x2QiQj,y3=y2QiQj (27)

To make it easier to express, we put forward a variable symbol *a*_*j*_:

$$a_{j}=\frac{Q_{i}}{Q_{j}}a_{i}$$ (28)

The horizontal span and height of the drag section:

$$x_{4}=a_{j}\;\text{arcsin}\;h(\frac{S_{4}}{a_{j}}),y_{4}=a_{j}(\text{cosh}\frac{x_{4}}{a_{j}}-1)$$ (29)

The horizontal span and height of the touchdown catenary section:

$$x_{5}=a_{i}\;\text{arcsin}\;h(\frac{S-S_{i}-S_{j}}{a_{i}}),y_{5}=\frac{y_{4}Q_{j}}{Q_{i}}$$ (30)

From the above equation, the height of the bending point B can be obtained:

$$y_{s}=y_{4}+y_{5}-y_{2}-y_{3}$$ (31)

The height of the arch point C can be expressed as:

$$y_{a}=y_{4}+y_{5}$$ (32)

Then the depth of the water can be obtained by the following equation:

$$V_{s}=y_{1}+y_{4}+y_{5}-y_{2}-y_{3}$$ (33)

The above equation can be used to check whether the structural equation of the LWR is calculated correctly.

### Contents flow effects

In OrcaFlex modeling, the pipe flow effect can generally be neglected. However, for pipes carrying high contents density at fast flow rates, the flow effects can be significant. In addition to gravity, pipe flow also brings three additional forces, including the centrifugal force, Coriolis force and liquid friction force.

First, the centrifugal force is calculated by considering a node with flow arriving from one direction and leaving in another direction. When the fluid passes through and leave a node, the unit vector of instantaneous direction is denoted as *μ*_*i*_ and *μ*_*o*_. In OrcaFlex, for the nodes between the segments of the pipeline, for a mid-node, *μ*_*i*_, *μ*_*o*_, are simply the unit vectors in the directions of the segments before and after the node. It is worth noting that *μ*_*i*_ and *μ*_*o*_ represent the same meaning for nodes whose ends are not constrained at both ends of the pipeline.

Therefore, the instantaneous velocity vector that flows into and out of a node can be expressed as *V*_*i*_*μ*_*i*_, *V*_*o*_*μ*_*o*_. The derivative of the momentum of the inflow part to the time is *ρS*_*i*_*V*_***i***_^***2***^*μ*_*i*_. Similarly, the derivative of the momentum of the outflow part to the time is *ρS*_*o*_*V*_***o***_^***2***^*μ*_***o***_. Thus, the centrifugal force acting on this node can be obtained:

$$F_{c1}=-\left(\rho S_{o}V_{o}{}^{2}\mu_{o\;-}\rho S_{i}V_{i}{}^{2}\mu_{i}\right)$$ (34)

Where, *F*_*c*1_ is the centrifugal force acting on the lumped mass nodes; *ρ* is the contents density; *S* is the internal cross-sectional area.

Since the motion of the pipeline segmentation leads to the generation of its internal liquid Coriolis force, the global coordinate system *G-xyz* and the local coordinate system *L-xyz* on the *N*_*i*_ node are used for the pipeline segment between the *N*_*i*_ and *N*_*i*_ + 1 nodes to describe the motion of its internal liquid, the vector *μ* is consistent with the direction from *N*_*i*_ towards *N*_*i*_+1.

The velocity expression in the global coordinate system can be obtained from the flow velocity of the liquid relative to the local coordinate system:

$$\nu_{L}=\left(q_{m}/S\rho \right)\mu$$ (35)

$$\nu_{G}=\nu_{i}+\left(q_{m}/S\rho \right)\mu +\omega \times \int \nu_{L}dt$$ (36)

In the global coordinate system, the acceleration can be expressed as:

$$a_{c}=\dot{\omega }\times \int \nu_{L}dt+2\omega \times \nu_{L}+\omega \times \nu_{i}+\omega \times (\omega \times \int \nu_{L}dt)$$ (37)

The second part of the expression is the acceleration component caused by Coriolis force, so the equation of Coriolis force can be expressed as the following:

$$F_{c2}=a_{c}m=2lq_{m}(\omega \times \mu )=2q_{m}\left(v_{i+1}-v_{i}\right)\text{sin}(\theta )$$ (38)

Where, *θ* represents the angle between the vector *v*_*i+1*_*-v*_*i*_ and the unit vector *μ*.

### Governing equation for the dynamic motion of the pipeline

In this paper, the dynamic analysis of the flexible riser is based on the classical Morison equation and Newton’s second law, in which the wave force is calculated by the Morison equation.

The Morison equation is first proposed by Morrison, as a special method to determine the vertical force of the cylinder. The Morison equation includes the effects of ideal fluids and viscous fluids.

When the diameter of the vertical cylinder is smaller than the wave length, the wave diffraction is relatively insignificant, and the effect of the viscosity is considered. In this case, the Morison equation can be used to obtain the cylindrical wave force. The original Morison equation develops into a formula on the horizontal wave force of a fixed vertical pipe and can be written as:

$$dF=\frac{1}{2}\rho_{\omega }C_{D}D\left|\dot{u}\right|\dot{u}+\frac{1}{4}\rho_{\omega }\pi D^{2}C_{M}\ddot{u}$$ (39)

Where, *dF* the wave force acting on the unit length of a vertical cylinder, $\dot{u}$ is the flow velocity, $\ddot{u}$ represents the horizontal acceleration of water particles; *ρ*_*ω*_ is the density of sea water; *D* is the diameter of the pipeline; *C*_*D*_ and *C*_*M*_ are the drag force coefficient and the inertia force coefficient.

If the vertical cylinder moves freely in the wave, the relative velocity and relative acceleration must be used in the Morison equation, and the extended form of Morison equation should be used:

$$dF=\frac{1}{2}\rho_{\omega }C_{D}D\left|{\dot{u}}_{n}-{\dot{u}}_{sn}\right|({\dot{u}}_{n}-{\dot{u}}_{sn})+\frac{1}{4}\rho_{\omega }\pi D^{2}\left[C_{M}{\ddot{u}}_{n}-(C_{M}-1){\ddot{u}}_{sn}\right]$$ (40)

Where, ${\dot{u}}_{sn}$ is the standard oscillation velocity of the pipeline; ${\ddot{u}}_{sn}$ is the standard oscillatory acceleration of the pipeline; $\dot{u}$ and $\ddot{u}$ are the standard velocity and acceleration of the pipeline, respectively.

The extended form of Morison’s equation is used in OrcaFlex. In the dynamic solution, the Lazy-wave risers are discretized into lumped mass models for solving the problem. The specific description of the lumped mass method can be referred to the reference [47–50].

The governing equation for the dynamic motion of the flexible risers which OrcaFlex solves is as follows:

M(s,a)+C(s,v)+K(s)=F(s,v,t) (41)

Where, *M*(*s*, *a*) is the inertia load of the flexible riser system; *C*(*s*, *v*) is the damping load of the flexible riser system; *K(s)* is the stiffness load of the flexible riser system; *F*(*s*, *v*, *t*) is the external excitation load of the flexible riser system; *s*, *v* and *a* are the position, velocity and acceleration of the flexible riser system, respectively; *t* is the simulation time.

Both schemes recompute the system geometry at every time step and so the simulation takes full account of all geometric non-linearities, including the spatial variation of both wave loads and contact loads.

### Motions of FPSO under the action of wave and 2nd order wave drift load

The ship wave frequency motion is calculated by response amplitude operator (RAO), and the 2nd order low frequency load can be solved by the full QTF matrix algorithm, and the response of the mooring line is solved with the ship motion.

For wave frequency motion, here we use the amplitude of response (in length units for surge, sway, heave, in degrees for roll, pitch, yaw) per unit wave amplitude, and to use the phase lag from the time the wave crest passes the RAO origin until the maximum positive excursion is reached (in other words, the phase origin being at the RAO origin). Mathematically, this is given by:

$$x=Ra\;\text{cos}\left(\omega t-\phi \right)$$ (42)

Where, *x* is the FPSO displacement (in length units for surge, sway, heave, in degrees for roll, pitch, yaw); *a*, *ω* are wave amplitude (in length units) and frequency (in radians/second), respectively; *t* is time (in seconds); *R*, *φ* are the *RAO* amplitude and phase.

At the same time, the FPSO motions with mooring systems can be described by the following:

$$\begin{array}{l} \left\{\left[M_{ij}\right]+[a_{ij}]\right\}\left\{{\ddot{x}}_{j}\right\}+\int_{-\infty }^{t}\left\{{\dot{x}}_{j}\right\}\left[R_{ij}(t-\tau )\right]d\tau +\left[C_{ij}\right]\left\{x_{j}\right\}\\ =\left[F_{wj}(t)\right]+\sum_{k=1}^{n}\left[F_{mk}(t)+F_{c}(t)+F_{wind}(t)\right] \end{array}$$ (43)

Where, *M*_*ij*_ is the mass, and *a*_*ij*_ is the added mass, *R*_*ij*_ is the time-memory function defined as following:

$$R_{ij}(t)=\frac{2}{\pi }\int_{0}^{\infty }B_{ij}(\omega )\frac{\text{sin}\;\omega t}{\omega }d\omega$$ (44)

Where, *B*_*ij*_ is the damping, *C*_*ij*_ is the restoring force determined by the FPSO and mooring system stiffness, *F*_*wj*_ is the wave force, including the first order and second order components, *F*_*mk*_ is the mooring force defined by the quasi-static method, *F*_*c*_ and *F*_*wind*_ are the current and wind forces.

The stiffness of the spread mooring system can be obtained by the catenary equations. The mass, damping, and restoring coefficients are determined by the linear potential theory. The second order non-linear force includes the mean and slow-drift wave force. The second slow-drift force can be written as:

$$\begin{array}{l} F_{i}^{SV}=\sum_{j=1}^{N}\sum_{k=1}^{N}A_{j}A_{k}\left[T_{jk}^{ic}\;\text{cos}\left\{\left(\omega_{k}-\omega_{j})t+(\varepsilon_{k}-\varepsilon_{j}\right)\right\}+T_{jk}^{is}\;\text{sin}\left\{\left(\omega_{k}-\omega_{j})t+(\varepsilon_{k}-\varepsilon_{j}\right)\right\}\right] \end{array}$$ (45)

Where, the coefficients $T_{jk}^{ic}$ and $T_{jk}^{is}$ can be interpreted as second-order transfer functions (QTF) for the different frequency wave loads.

In this paper, we used the hydrodynamic software AQWA to obtain the RAO of the FPSO and the 2nd order wave transfer function QTFs and then import them into OrcaFlex.

## Validation study

**Fig 5:**
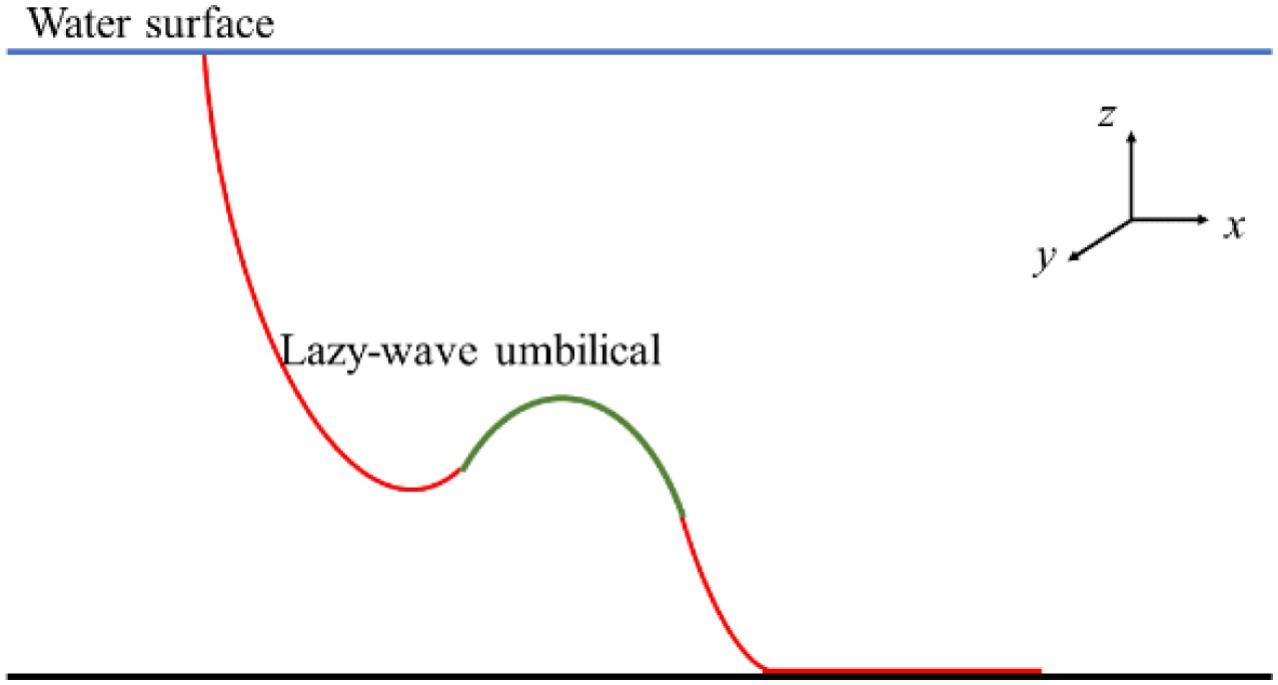
First validation model of OrcaFlex.

This paper focuses on the dynamic analysis of lazy-wave risers attached to FPSO, therefore, it is necessary to validate the correctness of numerical model for calculating the dynamic properties of the lazy-wave riser. To verify the reliability of the numerical model, a simulation of the nonlinear behavior of deepwater lazy-wave umbilical is carried out, as depicted in Fig 5. The OrcaFlex results, lazy-wave configuration, tension, bending moment and shear, are compared with the analytical results obtained from reference [26]. Basic properties of umbilical and environment are shown in Tables 1 and 2, respectively. Where, *w* and *w*_*e*_ are the submerged weights per unit length of decline section and buoyancy section, respectively; *L* and *L*_*e*_ are the lengths of decline section and buoyancy section, respectively; *D* and *D*_*e*_ are outer diameters of decline section and buoyancy section, respectively; *EI* and *EI*_*e*_ are the flexural stiffness of decline section and buoyancy section, respectively; *ρ* is the sea density. *k* is the elastic stiffness of the seabed; *v*_*c*_ is the current velocity; *C*_*d*_ and *C*_*τ*_ are the drag coefficient in the normal and tangential direction, respectively. The mechanical behavior of decline section is the same with hang-off section.

**Table 1:** The basic properties of the umbilical.

Property	Buoyancy section				Decline section				Axial stiffness EA (kN)
	*w*_*e*_ (N/m)	*L*_*e*_ (m)	*D*_*e*_ (m)	*EI*_*e*_ (kN.m^2^)	*w* (N/m)	*L* (m)	*D* (m)	*EI* (kN.m^2^)	
Value	-548.2	672	0.426	4.736×10^2^	501.7	1176	0.22	4.736×10^2^	1.652×10^6^

**Table 2:** Environmental parameters.

Parameter	Water depth(m)	*ρ*(kg/m^3^)	*k*(N/m^2^)	*v*_*c*_(m/s)	*θ*_0_(deg)	*C*_*d*_	*C*_*τ*_
Value	1500	1025	2×10^5^	0.2	87	0.7	0.008

**Fig 6:**
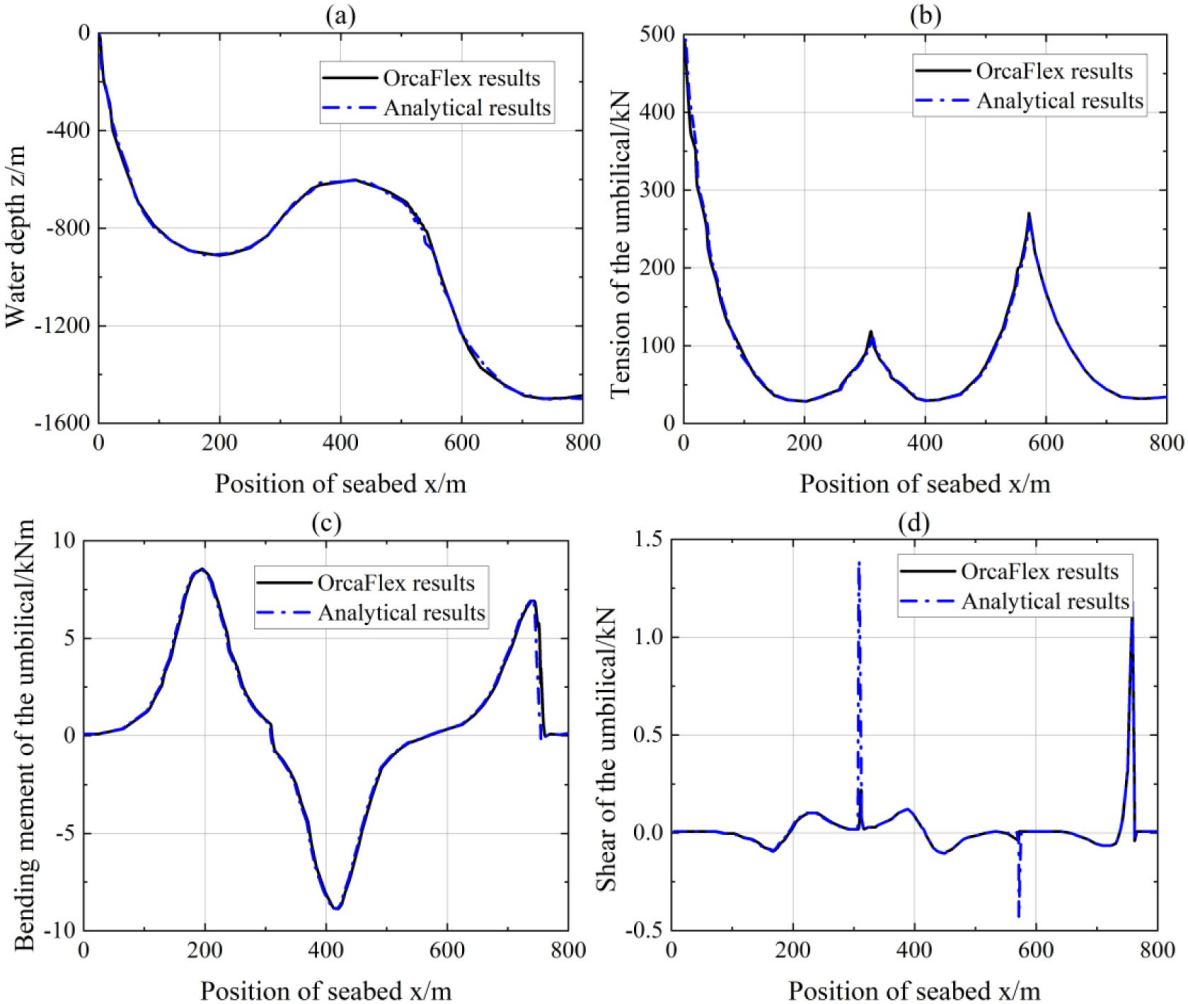
Comparison between the OrcaFlex results and analytical results [26]: (a) lazy-wave configurations; (b) lazy-wave tension; (c) lazy-wave bending moment; (d) lazy-wave shear.

As illustrated in Fig 6, the results obtained from OrcaFlex model and analytical model are in strong qualitative agreement. The comparisons can strongly verify the accuracy and reliability of the OrcaFlex model. In Fig 6(a), the lazy-wave configurations of OrcaFlex results are in coincidence with the analytical results, except for the x position around 550m, which is in the end of the buoyancy section. The reason may be that the submerged weight per unit length and diameter of the umbilical cable have changed from the buoyancy section to the decline section, resulting in calculation errors. However, the difference is still less than 5%. Fig 6(b) also depicts the good consistency between two methods. It can be found that three local maximum tensions occur at the hang-off point, lift point and decline point respectively. Among the three local maximum tensions, the tension at the hang-off point seems to be the maximum, which is far less than the hang-off tension 915.7 kN in simple free hanging configuration under the same condition. Fig 6(c) shows the comparison of umbilical’s bending moments. The overall bending moments almost perfectly match, and the main difference shows in the neighborhood of the touchdown point. Notably, the shear of analytical model occurs two peaks at around 300m and 560m. This is because the flexural stiffness is considered in the boundary-layer segment, but not considered in the decline section.

## Establishment of the model

The compositions and layouts of the riser system are shown in Table 3.

**Table 3:** The parameters of the 55 Lazy-wave risers.

Number	Type	Dimension(inch)	Contents	Direction	Layout angle(°)	Hanging off angle(°)
**1**	SLWR	6	Water	Port	-34.0	7
**2**	SLWR	4	Water	Port	-31.7	7
**3**	SLWR	6	Oil	Port	-29.4	7
**4**	SLWR	4	Water	Port	-27.1	7
**5**	SLWR	6	Water	Port	-24.8	7
**6**	SLWR	6	Oil	Port	-22.5	7
**7**	SLWR	6	Gas	Port	-19.5	7
**8**	SLWR	6	Water	Port	-16.5	7
**9**	SLWR	6	Oil	Port	-14.2	7
**10**	SLWR	6	Water	Starboard	-28.0	7
**11**	SLWR	6	Oil	Starboard	-25.8	7
**12**	SLWR	4	Water	Starboard	-23.7	7
**13**	SLWR	6	Water	Starboard	-21.5	7
**14**	SLWR	6	Water	Starboard	-19.3	7
**15**	SLWR	6	Water	Starboard	-17.1	7
**16**	SLWR	6	Water	Starboard	-14.9	7
**17**	SLWR	4	Water	Starboard	-12.8	7
**18**	SLWR	6	Oil	Starboard	-10.6	7
**19**	SLWR	6	Water	Starboard	-8.4	7
**20**	SLWR	4	Water	Starboard	-6.3	7
**21**	SLWR	6	Gas	Port	-11.0	7
**22**	SLWR	6	Water	Port	-9.0	7
**23**	SLWR	6	Oil	Port	-7.0	7
**24**	SLWR	4	Water	Port	-5.0	7
**25**	SLWR	6	Water	Port	-3.0	7
**26**	SLWR	6	Oil	Port	-1.0	7
**27**	SLWR	6	Water	Starboard	-4.0	7
**28**	SLWR	6	Gas	Starboard	-1.9	7
**29**	SLWR	6	Oil	Starboard	-0.3	7
**30**	SLWR	6	Water	Starboard	-2.4	7
**31**	SLWR	6	Gas	Starboard	5.4	7
**32**	SLWR	6	Oil	Starboard	8.4	7
**33**	SLWR	6	Water	Starboard	10.6	7
**34**	SLWR	4	Water	Starboard	12.8	7
**35**	SLWR	6	Oil	Starboard	15.0	7
**36**	SLWR	6	Water	Starboard	17.1	7
**37**	SLWR	4	Water	Starboard	19.3	7
**38**	SLWR	6	Oil	Starboard	21.5	7
**39**	SLWR	6	Water	Starboard	23.7	7
**40**	SLWR	4	Water	Starboard	25.8	7
**41**	SLWR	6	Water	Starboard	28.0	7
**42**	SLWR	6	Gas	Port	5.0	7
**43**	SLWR	6	Water	Port	7.0	7
**44**	SLWR	6	Oil	Port	9.3	7
**45**	SLWR	4	Water	Port	11.6	7
**46**	SLWR	6	Water	Port	13.9	7
**47**	SLWR	6	Oil	Port	16.2	7
**48**	SLWR	4	Water	Port	18.5	7
**49**	SLWR	6	Water	Port	20.8	7
**50**	SLWR	6	Oil	Port	23.1	7
**51**	SLWR	6	Water	Port	25.4	7
**52**	SLWR	6	Water	Port	27.7	7
**53**	SLWR	6	Gas	Port	30.7	7
**54**	SLWR	6	Water	Port	33.7	7
**55**	SLWR	6	Water	Port	36.0	7

SLWR is the abbreviation of Steel Lazy-wave riser.

**Fig 7:**
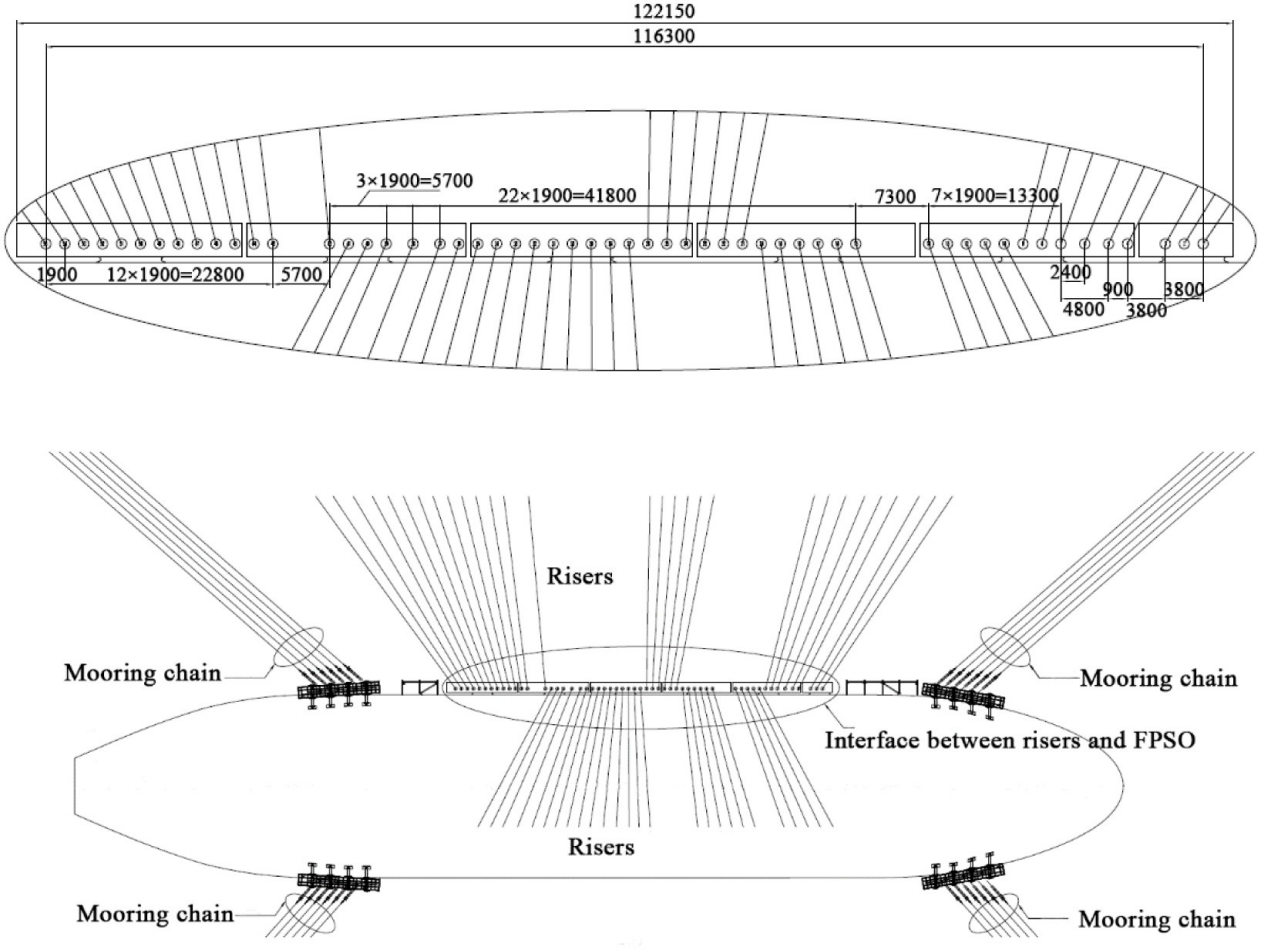
General layout of risers and mooring cables.

The length of each single riser is 3673.71m. According to the above theory, the floating buoy is added at different positions to make the configuration in the form of Lazy wave. At the same time, the hydrodynamic coefficients of each segment are equivalent to ensure the equivalence of the hydrodynamic performance in the analysis process. When the content flow is water, the internal pressure is 9997.7kPa and the temperature is 25°, the flow density is 1.025t/m^3^; when the content flow is oil, the internal pressure is 34475kPa and the temperature is 100°, the flow density is 0.8009t/m^3^; when the content flow is oil, the internal pressure is 34475kPa and the temperature is 100°, the flow density is 0.2403t/m^3^. The sea bed is flat and uniform. The general layout of risers and mooring cables is shown in Fig 7.

The whole system is moored by 24 mooring cables. Due to the different layout positions of the mooring cables, the length of each mooring cable is between 4250m and 4315m. The axial stiffness of the cable *EA* is 6000KN. The bending stiffness *EI* is 0. Each mooring line is made of a blend of materials, the middle section is a certain length of steel cable, the other sections are the equivalent buoyant cables.

The length of FPSO is 319m, the breadth is 58m, the moulded depth is 31m, the total tonnage is 360 thousand tons; the moment of inertia is *I*_*x*_ = 126.5×10^6^ t.m^2^, *I*_*y*_ = 300.7×10^6^ t.m^2^, *I*_*z*_ = 307.5×10^6^ t.m^2^, respectively. The hydrodynamic coefficients of FPSO, RAO, QTFs can be obtained by AQWA [51] and then they will be imported into OrcaFlex [52].

The calculation environment condition is chosen as the 100 years working condition in Table 4. The significant wave height H_s_ and the maximum wave height H_max_ are 4.5m and 7.8m, respectively. The spectral peak period T_p_ and the zero-crossing period T_z_ are 10.3s and 7.3s. The current velocities of surface layer, intermediate layer and bottom layer are 1.2ft/s, 0.64ft/s and 0.52ft/s, respectively.

**Table 4:** The working conditions.

	Parameters	Return period					
		1year	5years	10 years	25 years	50 years	100 years
**Wave**	H_s_(m)	2.8	3.5	3.8	4.2	4.3	4.5
	H_max_(m)	4.9	6	6.6	7.2	7.5	7.8
	T_z_(s)	6	6.5	6.8	7	7.2	7.3
	T_p_(s)	8.4	9.2	9.6	9.9	10.1	10.3
**Current(ft/s)**	Surface layer	0.78	0.91	0.99	1.08	1.14	1.2
	Intermediate layer	0.45	0.48	0.52	0.57	0.61	0.64
	Bottom layer	0.43	0.46	0.47	0.49	0.5	0.52

As the large inclination instability of is prone to occur when it is in the beam waves, therefore, the calculation condition of 100 years return period is selected for the simulation and in the process of modeling, the beam wave means the wave direction and current direction are all 90°. As what we need here is the coupling analysis of risers and FPSO, not the strength analysis or fatigue analysis. This analysis is to obtain the overall dynamic response when the system reaches a steady state, so there is no need to set the simulation time too long, as long as the system is stable in a certain simulation period, the overall response characteristics of the system can be displayed. And the total system flexible components reach 79, if the simulation time is set to 3 hours according to the requirement of marine engineering, it will take a large amount of calculation resources and consume a large amount of calculation time, which is extremely uneconomical. After many times debugging, it is found that the whole system has entered a stable overall response stage after 100s. Therefore, the dynamic simulation stage is set to 200s, and the static analysis stage is 20s, so the setting is enough to ensure the dynamic response of the system to be stable in this time period.

In this simulation, the time of dynamic calculation is proportional to product between the number of nodes and the built-in time steps; if OrcaFlex default time step is used and the nodes are evenly divided, then the computation time is approximately proportional to the cube of the node number. In that case, the more nodes there are, the more time it takes to calculate. In fact, there is no need for that. For some parts with serious bending, friction, frequent contact and possible stress concentration, it is necessary to divide the segments into more dense nodes; for some parts which have relatively simple spatial configuration and are far away from the top of the hanging off segments, the nodes can be segmented more sparsely, so that the calculation can meet the requirements of the project and save a lot of computing time.

**Fig 8:**
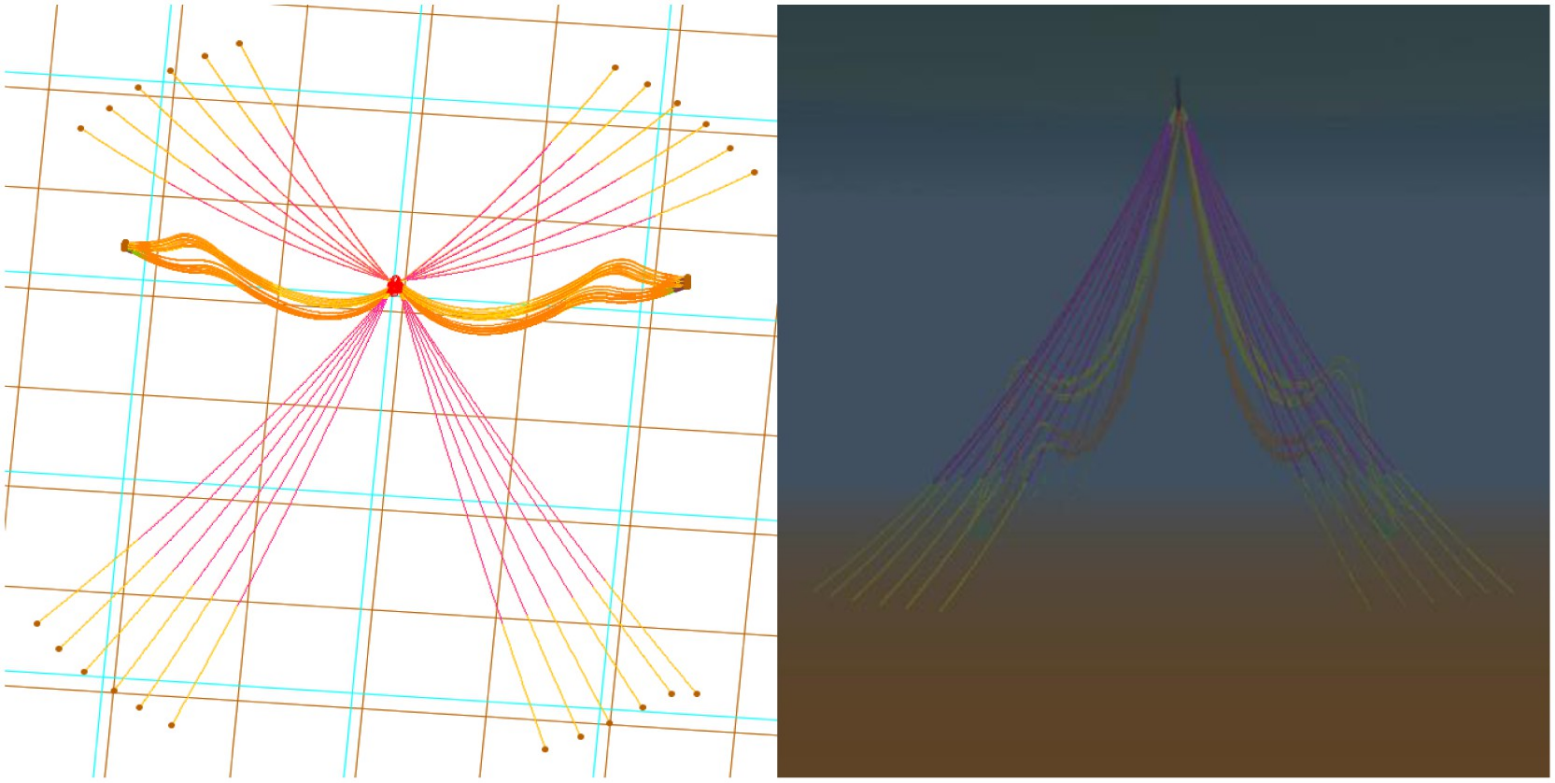
The model in OrcaFlex.

The model of the system completed in OrcaFlex is as shown in Fig 8.

## Results and discussion

### Calculation results without 2nd order wave drift loads

**Fig 9:**
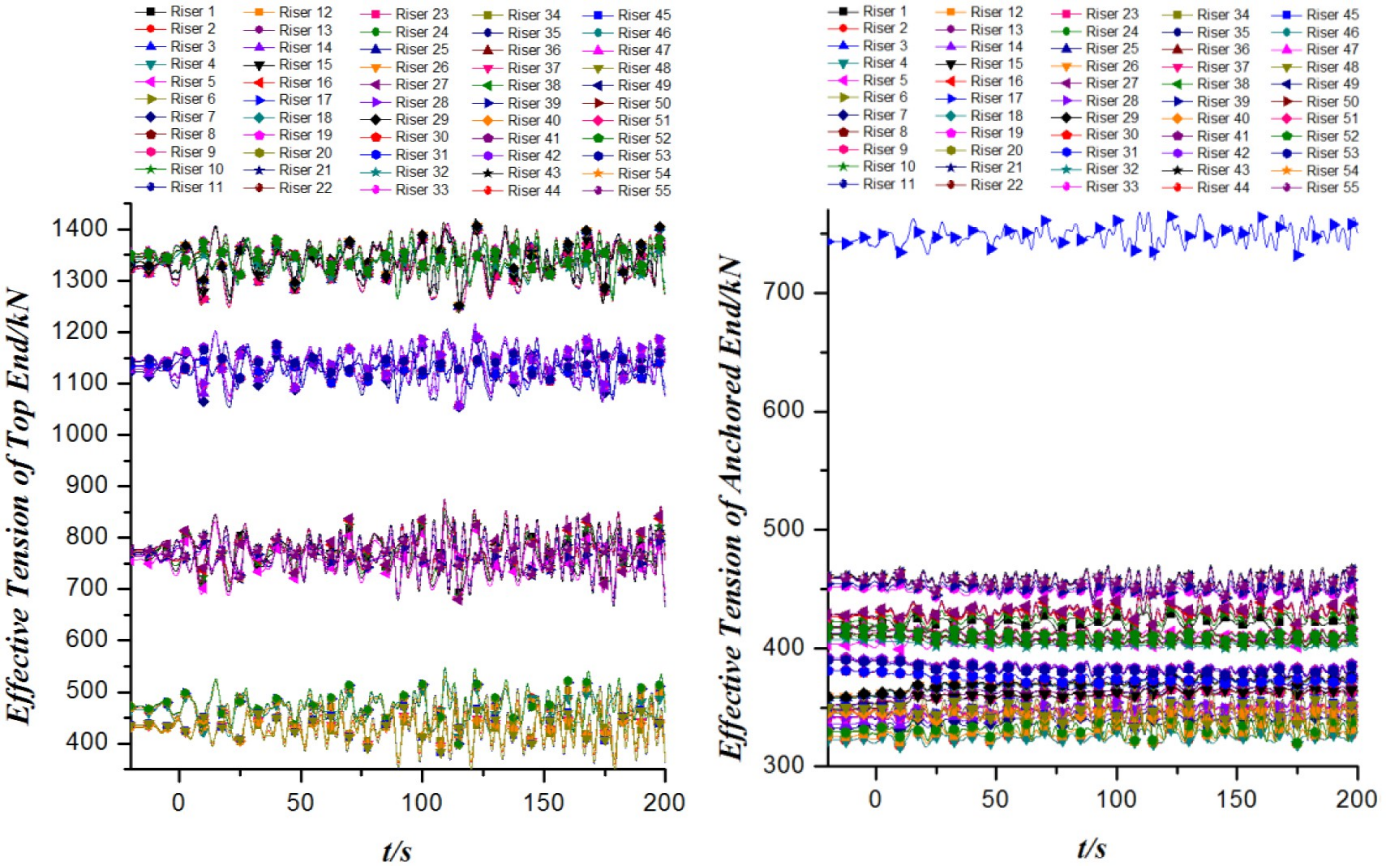
The effective tension of 55 risers versus time.

Fig 9 depicts the effective tension of 55 risers. It is found that the curves of the top end tension of the risers whose coordinate values are close on the FPSO are highly coincident. As the layout of the risers on the FPSO is generally near the starboard and port sides of the bow and the starboard side of the stern. Therefore, the top tension curves of different risers coincide highly with 4 different curves in the time domain. In contrast to the top tension curves, the curves of the effective tension of the anchored ends of the 55 risers have a strong similarity in the time domain. In particular, the tension value of the anchored end of the Riser 17 is far larger than that of the anchored end of the other risers.

**Fig 10:**
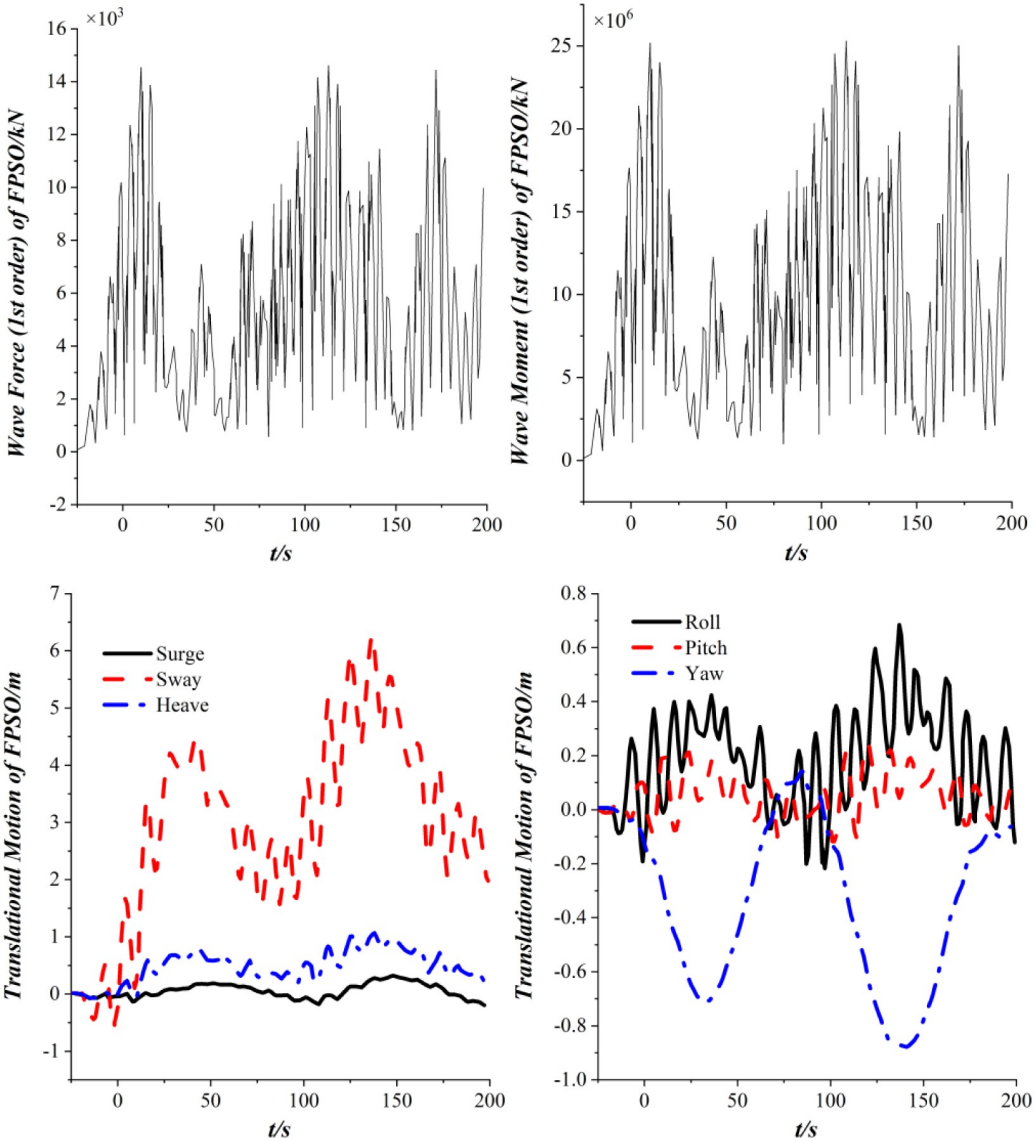
Dynamic response of the FPSO.

Fig 10 shows the dynamic response of the FPSO in the time domain. Fig 8 shows that the first-order wave forces and moments acting on FPSO exhibit periodic changes in the time domain when only the first-order wave load is considered. And the amplitude of sway in the translational motion of FPSO is the largest, the amplitude of surge and heave is small, and the amplitude of the roll is the largest in the rotational motion of the FPSO. Further observation reveals that relatively high-frequency vibration occurs in sway and roll, due to the layout between the FPSO and the wave direction.

**Fig 11:**
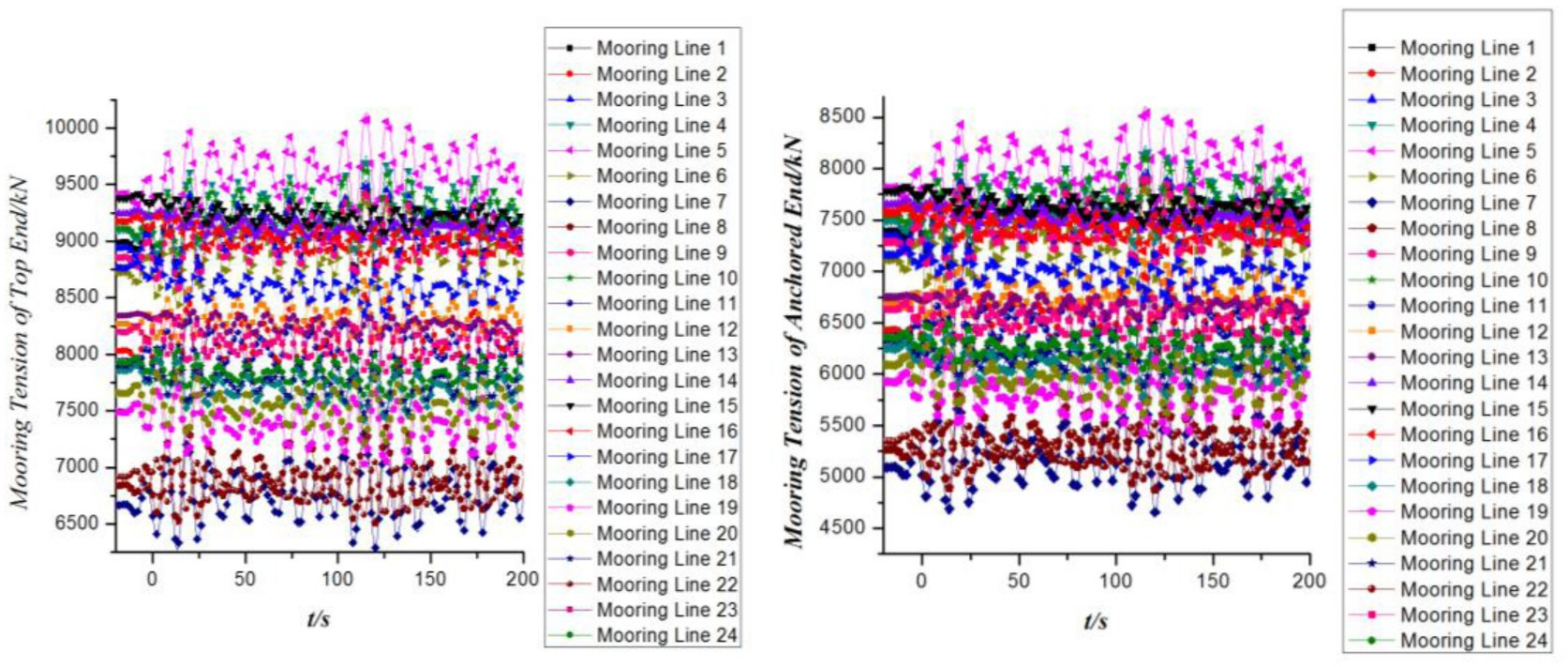
Mooring tension of mooring cables versus time.

The time domain curves of mooring tension at different ends of the mooring cables are observed in Fig 11. In general, the mooring tension at the top end of all mooring lines is greater than that of the anchored ends. As the layout of the mooring cables on the FPSO is generally near the starboard and port sides of the bow and the starboard side of the stern. Therefore, the mooring tension curves of different mooring cables coincide highly with 4 different curves in the time domain. In addition, the configurations of the curves of the mooring tension at the top and the anchored ends are basically the same except that the numerical values are different.

### Calculation results with 2nd order wave drift loads

**Fig 12:**
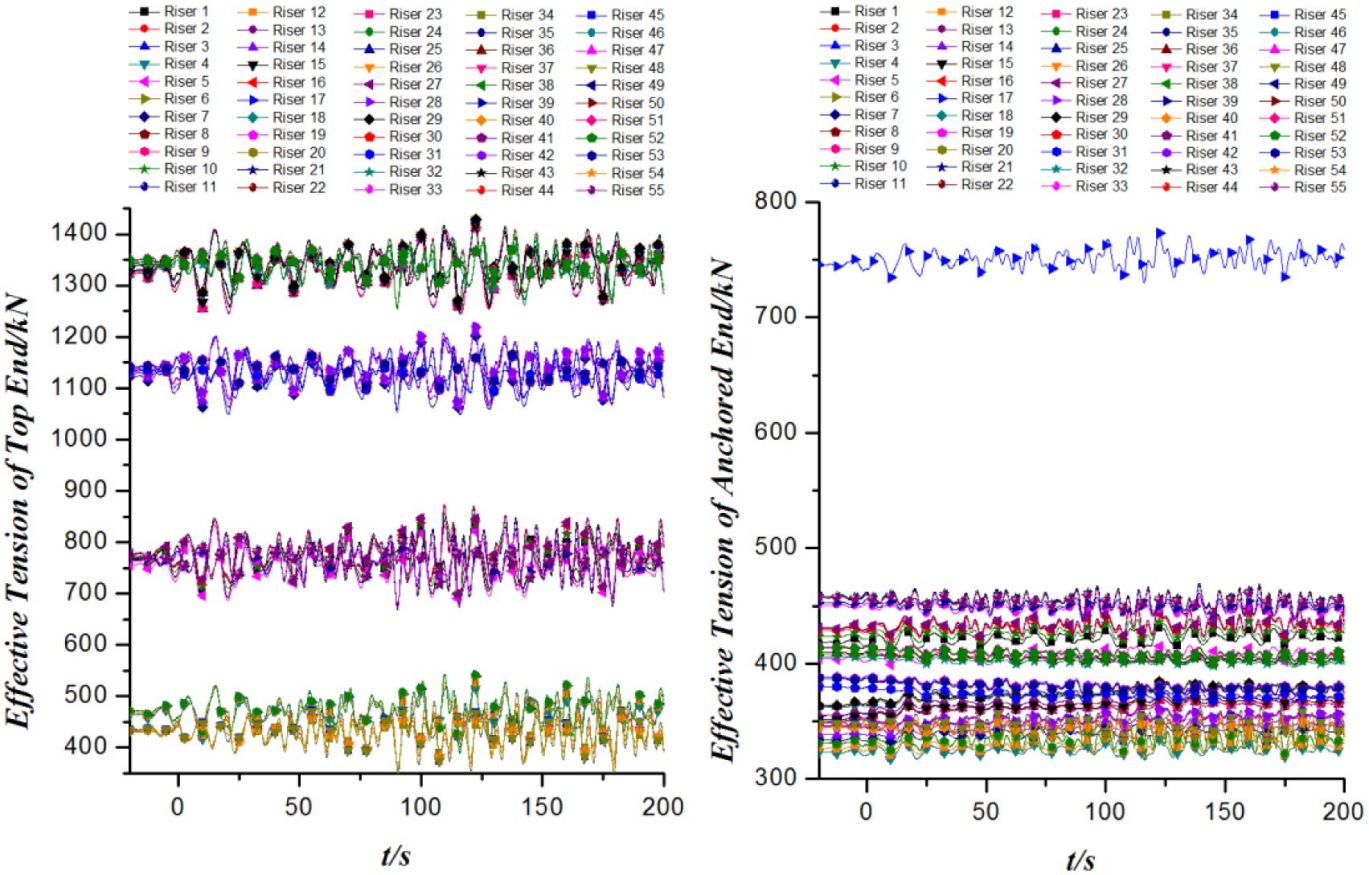
Effective tension of mooring cables versus time.

With the comparison of Figs 9 and 12, it is found that the effective tension at each end of each riser increases to some extent when considering the 2nd order wave drift loads. Among them, the increase of the effective tension of Riser 17 at the anchored end is particularly evident. Here, it should be pointed out that the excessive tension of Riser17 at the anchored end can easily cause local radial shrinkage in the radial direction of the pipeline, which can lead to oil pipeline fracture and oil spill. Once the oil spill happens, it will have a very bad impact on the environment. Therefore, the oil spill risk assessment and measures for the anchored end of Riser 17 should be taken to avoid the pollution of the sea water.

**Fig 13:**
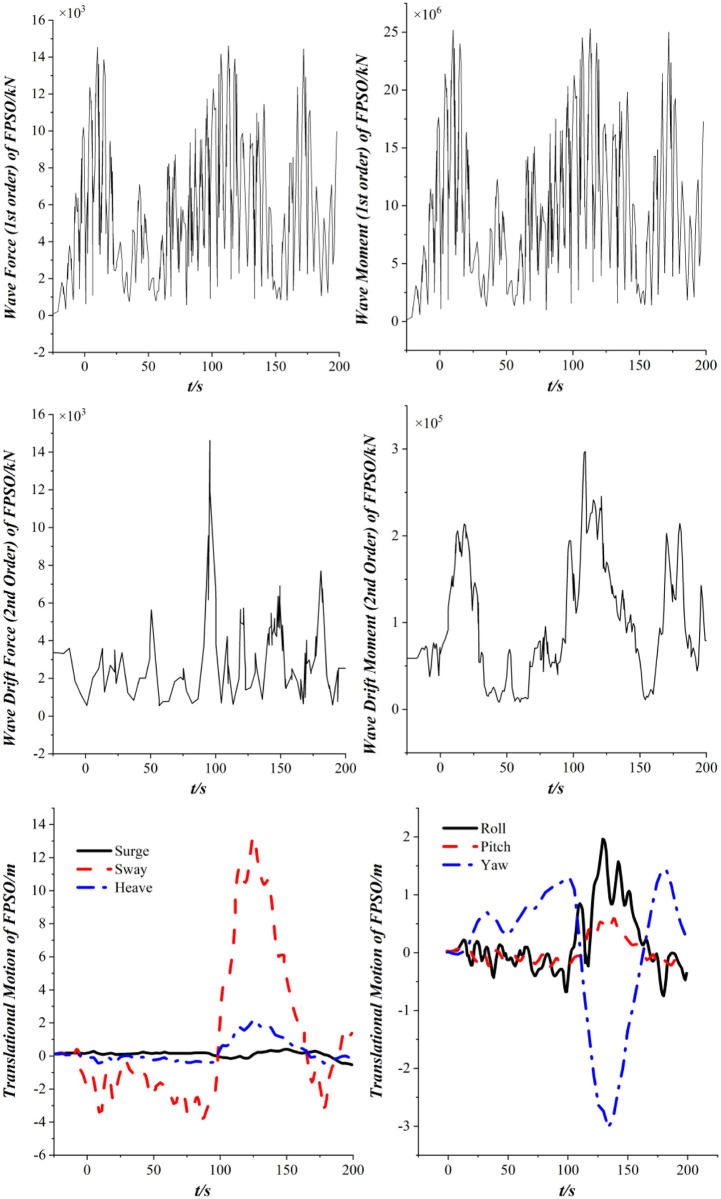
Mooring tension of mooring cables versus time.

Fig 13 shows the dynamic response of the FPSO in the time domain when the 2nd order wave drift loads are taken into account. It indicates that with the consideration of the 2nd order wave drift loads, the amplitude sway of the FPSO amplitude increases significantly, and the amplitude of roll and yaw increases greatly, and the yaw angle has recurrent fluctuations in the positive and negative directions. The motion amplitude of FPSO in the direction of sway is far higher than that of the surge direction. As the influence of the 2nd order wave drift loads is considered in the calculation of ship motion. The 2nd order wave drift loads affect the coupling degree of each degree of freedom of the FPSO, which is reflected in the change of tension distribution of the mooring cables and risers.

**Fig 14:**
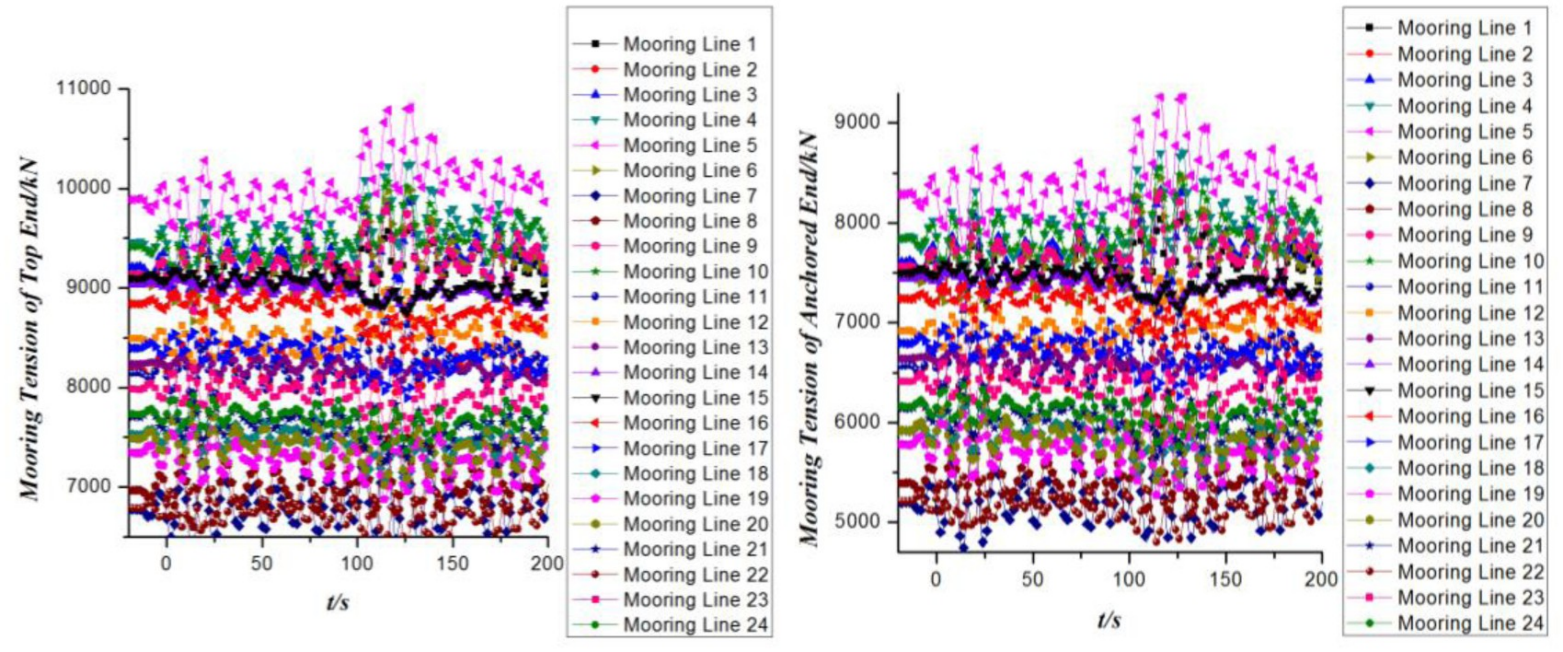
Mooring tension of mooring cables versus time.

Fig 14 shows the dynamic response of the mooring tension in the time domain when the 2nd order wave drift loads are taken into account. It indicates that the mooring tension at both ends of the mooring cables increases greatly when the 2nd order wave drift loads are taken into consideration. Compared with the risers, the mooring system takes on more tension loads regardless of whether the two order wave forces are considered.

## Conclusions

For the underwater production system with large displacement and large number of system flexible components which is running in ultra-deep water areas, it is a great challenge to make simulation convergence The dynamic characteristics of a subsea production system with 55 Lazy-wave risers is investigated. A simplified dynamic model of the whole system at the depth of 2100m has been established by the large hydrodynamic analysis software OrcaFlex. Besides, the effects of the 2nd wave drift load on the results have been obtained. This study demonstrated the following:

1. The huge number of flexible components and risers increases the difficulty the convergence of the system. The rigid-flexible coupling between the FPSO which is seemed as a rigid body and the pipelines which is seemed as flexible members happens, the motions of the FPSO are limited by the risers and mooring cables and the response of the riser and mooring lines is also affected by the hull movement, it makes searching for static balance of the whole system difficult. And it is a very huge challenge to make the convergence of the model.
2. The simulation results show that the 2nd order wave drift loads have a significant effect on the riser system. When considering 2nd order wave drift loads, the six DOF of the FPSO is more complex and coupled, and the tension of the risers and mooring cables also increases. The amplitude sway of the FPSO amplitude increases significantly, and the amplitude of roll and yaw increases greatly, and the yaw angle has recurrent fluctuations in the positive and negative directions.
3. For the complex riser system which is composed of more flexible components, the 2nd wave drift loads must be considered. To counteract the magnitude of the FPSO response caused by such loads, the number of mooring lines needed to be increased or combined with dynamic positioning techniques to optimize the design. However, for such a huge FPSO, the use of dynamic positioning technology will undoubtedly greatly increase the cost.

Notable, the effective tension of most risers is not significantly different, however, only one riser has a significantly higher effective tension than the other risers. This may be related to its layout angle and hang off angle. Further research and exploration of the underlying causes are worth pursuing.

## Supporting information

S1 Dataset (XLSX)

S1 Data (XLSX)

S2 Data (XLSX)

S3 Data (XLSX)

S4 Data (XLSX)

S5 Data (XLSX)
